# Improvement of Plasterboard Properties by the Control of Polymethylhydrosiloxane Dosage, Stirring Time, and Drying Temperature Applied to the Calcium Sulfate Hemihydrate and Water Mixture

**DOI:** 10.3390/ma16145084

**Published:** 2023-07-19

**Authors:** Victoria Romano-Matos, Alain Tundidor-Camba, Sergio Vera, Alvaro R. Videla

**Affiliations:** 1 Departamento de Minería, Escuela de Ingeniería, Pontificia Universidad Católica de Chile, Santiago 7820436, Chile; vromano@uc.cl; 2 Facultad de Química y Farmacia, Pontificia Universidad Católica de Chile, Santiago 7820436, Chile; atundido@uc.cl; 3 Research Laboratory for Organic Polymers (RLOP), Department of Organic Chemistry, Pontificia Universidad Católica de Chile, Santiago 7820436, Chile; 4 Departamento de Ingeniería y Gestión de la Construcción, Escuela de Ingeniería, Pontificia Universidad Católica de Chile, Santiago 7820436, Chile; svera@ing.puc.cl; 5 Center for Sustainable Urban Development CEDEUS, Pontificia Universidad Católica de Chile, Santiago 7820436, Chile; 6 Centro de Energía UC, Escuela de Ingeniería, Pontificia Universidad Católica de Chile, Santiago 7820436, Chile

**Keywords:** polymethylhydrosiloxane (PMHS), plasterboard, water absorption, flexural strength, thermal conductivity, morphology, porosity, X-ray microcomputed tomography (XMT), scanning electron microscopy (SEM), dihydrate, hemihydrate

## Abstract

Plasterboard is an important building material in the construction industry because it allows for quick installation of walls, partitions, and ceilings. Although a common material, knowledge about its performance related to modern polymers and fabrication conditions is still lacking. The present work analyzes how some manufacturing factors applied during the plaster board fabrication impact on some plasterboard properties, including water absorption, flexural strength, and thermal conductivity. The manufacturing variables evaluated are the dose (D) of polymethylhydrosiloxane (PMHS), the agitation time of the mixture (H), and the drying temperature of the plaster boards after setting (T). The results suggest that factors D, H, and T induce changes in the porosity and the morphological structure of the calcium sulfate dihydrate crystals formed. Performance is evaluated at two levels of each factor following a statistical method of factorial experimental design centered on a cube. Morphological changes in the crystals of the resulting boards were evaluated with scanning electron microscopy (SEM) and the IMAGEJ image analysis program. Porosity changes were evaluated with X-ray microcomputed tomography (XMT) and 3D image analysis tools. The length-to-width ratio of the crystals decreases as it goes from low PMHS dosage to high dosage, favoring a better compaction of the plasterboard under the right stirring time and drying temperature. In contrast, the porosity generated by the incorporation of PMHS increases when going from low-level to high-level conditions and affects the maximum size of the pores being generated, with a maximum value achieved at 0.6% dosage, 40 s, and 140 °C conditions. The presence of an optimal PMHS dosage value that is approximately 0.6–1.0% is evidenced. In fact, when comparing trails without and with PMHS addition, a 10% decrease in thermal conductivity is achieved at high H (60 s) and high T (150 °C) level conditions. Water absorption decreases by more than 90% when PMHS is added, mainly due to the hydrophobic action of the PMHS. Minimum water absorption levels can be obtained at high drying temperatures. Finally, the resistance to flexion is not affected by the addition of PMHS because apparently there are two opposing forces acting: on one hand is the decrease in the length–width ratio giving more compactness, and on the other hand is the generation of pores. The maximum resistance to flexion was found around a dosage of 0.6% PMHS. In conclusion, the results suggest that the addition of PMHS, the correct agitation time of the mixture, and the drying temperature reduce the water absorption and the thermal conductivity of the gypsum boards, with no significant changes in the flexural resistance.

## 1. Introduction

Natural gypsum, or calcium sulfate dihydrate (DH), is widely used as a building material in diverse types of products. Laminated plasterboards are among the most used products in the world due to their low cost, light weight, and ease of installation. They are used for walls, internal partitions, and ceilings in buildings. 

Plasterboard manufacturing involves several stages. First, DH must be dehydrated, causing a mineralogical change from DH to HH (hemihydrate). HH is mixed with water to generate a homogeneous paste or grout, which is then poured onto cardboard with a defined dimension that is molded. The board is set for hardening and finally dried to remove excess water. 

Plasterboard comprises a hardened DH center, which is laminated on both sides with paper. Boards are compact and solid but consist of rather highly porous material whose internal surface is made up of interlocking crystals in the form of plates and needles whose microstructure affects most of the final product’s physical properties [1]. High-pore content, water solubility, and relatively large crystals contribute greatly to water affinity [2].

One of the key plasterboard manufacturing stages relates to the change of HH to DH. The HH species is less stable than DH, so upon contact with water a rearrangement of the water molecules occurs with sulfate and calcium ions, forming the DH species, which is more stable. This is known as a hydration reaction. Some researchers, such as Singh and Middendorf [1] and Cheng et al. [3], agree that HH hydration leading to DH formation occurs through a dissolution mechanism, i.e., HH particles dissolve in water and then DH precipitates from the aqueous solution by being less soluble than HH [4,5,6,7]. Using smaller HH particles ensures rapid dissolution before setting begins, which prevents the formation of mesopores [8]. Crystal size changes depend on agitation time during DH crystallization [9]. However, these studies focused on pure gypsum and did not incorporate chemical additives that could affect crystallization and morphology.

Some studies [10,11] have shown that the addition of chemical additives may allow better control of the final morphological properties resulting from the production of DH from HH. In fact, it has been reported that a hydrophobic, water-repellent organic emulsion could coat the HH particle while hydrating, inducing a change of structure and shape of the hydration products. As a result, the surface of the hydration products becomes smooth, preventing further hydration, and the needle-shaped interwoven DH crystals gradually grow to form a thick, short column on the matrix. Regarding the interactions between HH and additives, Nicoleau et al. [12] has shown that nucleation groups are formed by the union of primary species, where the collision probability is reduced in the presence of an additive that interacts with the species, leading to more time to form a critical nucleus capable of maturing in crystalline gypsum. There are several investigations on the effects that additives cause to the hydration reaction; for example, some have shown how the presence of an additive in the reaction improved the agglomeration of crystals, decreased the total volume of pores, and increased the overlapping of crystals, leading to a higher mechanical resistance [13,14,15]. In another investigation, two additives were added to the hydration reaction, with one of them leading to a reduction of the mechanical resistance. This effect was attributed to the change in morphology from needle to column or plate type and higher porosity [16]. In the same study, the best mechanical resistance was attributed to the formation of a greater number of intertwined needle-shaped crystals. Additional investigations can be found with evaluations of additives and their effects on the final morphology of the formed plaster and its properties [17,18,19,20,21,22,23,24]. 

Pan and Li [25] proposed improving moisture resistance by using a mixture containing fluorinated silicone as an additive. The film formed by the impermeable agent coats the microscopic pore, changing the energy of the macropores’ inner surface from hydrophilic to hydrophobic, thereby repelling water and achieving greater water resistance by preventing the access of a drop or increased water penetration into the matrix. Wang, Cui, and Xue [26] found that methyl sodium silanol reduced pore size from 500 microns to 100 microns on average by increasing the number of connected pores, causing an increase in water absorption of the sample. Studies have shown that some additives accelerate hydration and lead to the formation of a dense and well-compacted morphological texture, which imparts better moisture resistance onto the gypsum matrix [27,28,29,30]. Previous studies also show that additive type and amount influence the hydration process, and the final DH structure could produce considerably higher porosity, so additives are used to generate fine structures related to better mechanical strength and moisture resistance [27,31,32,33,34].

On the other hand, the polymer polymethylhydrosiloxane (PMHS) is used as a reducing agent and has aroused interest because of its hydrophobic potential. The PMHS repeating unit comprises Si, O, and H atoms and covalently linked methyl groups. The methyl groups give it a highly hydrophobic character, which can be verified using a Krüss drop-shaped analysis system, which allows its contact angle measurement, reaching the order of 100 ± 2.0° on the plaster [35,36,37]. Keller evaluated the effect of PMHS as a hydrophobic agent in doses between 0.4% and 1.0% of weight [38]. First, the PMHS is dispersed in the water and the hydrolysis reaction occurs, forming silanol (SiOH), which is adsorbed on the crystal surfaces, then the condensation reaction occurs, two silanol molecules react releasing water molecule and forming the Si-O-Si bond on the crystal surfaces with the methyl groups directed away from the crystal surfaces, and as they are hydrophobic they repel water [39,40,41]. The effect of PMHS in gypsum has been studied on waterproof blocks that were prepared with flue gas desulfurization gypsum (FDG) mixed with fly ash, sodium sulfate, and PMHS. An addition of 0.4% PMHS achieved a water absorption of 3.25%, explaining how PMHS affects the morphology of DH crystals and thus, at the same time, the water absorption property. Additionally, there are patents on how to make gypsum-based products, such as plasterboard, with water repellent properties using silicone additive mixtures [41,42,43,44].

Previous investigations did not consider linking the use of PMHS and the combined effect of production operational manufacturing variables with the final properties of gypsum plasterboard. A better understanding could improve the performance and extend the applications of plasterboard to several new uses due to its low cost. Therefore, this investigation focuses on the effects of the PMHS dosage, agitation time, and drying temperature on the morphology of the crystals and the porosity of the gypsum boards, and how these effects translate into changes in properties, water absorption, flexural strength, and thermal conductivity.

## 2. Materials and Methods

### 2.1. Materials and Equipment

Beta-calcium sulfate hemihydrate was used for the study, which is a commercial calcium sulfate hemihydrate used for construction and complies with Chilean standard NCh 143 [45]. In addition, PMHS from Elkem Silicones France Bluesil WR 68 was used.

The X-ray fluorescence (XRF) WDX S4 sequential spectrometer, dispersive in wavelength, was used to discern the chemical composition of calcium sulfate hemihydrate. Samples were calcined at a temperature of 1050 °C for two and a half hours to obtain all compounds in their oxidation state. Additionally, a homogeneous vitreous disc was obtained via the fusion method, using an M4 Claisse fusion unit.

A Bruker D8 Advance diffractometer was used to determine the mineralogical composition of calcium sulfate hemihydrate. The powdered samples were placed in the sample holders and gently pressed.

The morphology of calcium sulfate hemihydrate crystals and calcium sulfate dihydrate crystals was determined with SEM device FESEM-EDS FEI QUANTA FEG 250. After taking the images, the length–width ratio of calcium sulfate dihydrate crystals was determined by processing the images with IMAGEJ software v1.53k.

The particle size distribution of HH and DH was analyzed with a Mastersizer 2000, which measures particle size by scattering laser beams in liquid suspension, using a laser diffraction analyzer (LDA). Isopropyl alcohol was used with a particle concentration of 0.0119 vol%, which achieved a Particle RI of 1.52 and Opacity of 17.15%.

The main functional groups present in the PMHS additive and in the DH samples with PMHS were analyzed with a Shimadzu infrared spectrometer (FTIR), Model IRTracer 100.

As indicated by Capasso et al. [34] and others [46,47,48], the pore dispersion was measured with X-ray microcomputed tomography, which allows one to explore the interior of solid samples by means of 3D digital image reconstruction.

A high-resolution SkyScan Bruker 1272 device was used at 80 kV, 125 mA, with a rotation pitch of 0.4°, using a 0.25 mm aluminum filter and a voxel size of 12 μm resolution. Three-dimensional scanned images were obtained with the NRecom reconstruction software. The images were reoriented in space using the DataViewer software v1.5.1.9 to standardize sample positioning. Within a volume of interest (VOI) in the transverse plane of approximately 2 cm^3^, quantitative evaluation was conducted using CT and software analysis. Finally, images and videos of each sample were obtained using visualization software. Pixel size was 24 μm. Voxels in the 3D image with gray scale values between 25 and 255 were considered solid material, while voxels with values between 24 and zero were taken as void space.

### 2.2. Experimental Methodology

In this investigation, PMHS, HH, and water were used for material preparation. According to the factorial experimental design of the investigation, independent variables or factors were considered, in which each factor varied at defined levels. In this investigation, the factors selected were the PMHS dose (D), the agitation time (H), and the drying temperature (T). The levels considered for each factor were:

D: minimum 0.2%, maximum 1.0%, midpoint 0.6%;
H: minimum 20 s, maximum 60 s, midpoint 40 s;
T: minimum 130 °C, maximum 150 °C, midpoint 140 °C.

We have considered a cube-centered factorial design with the aim of achieving a possible optimal value. According to the factorial experimental design centered on a cube, the number of different mixtures to perform is given by the ratio of the number of levels raised to the number of factors plus the midpoint. Thus, in the case of three factors, it would be 2^3^, which would be 8 plus the central point, giving a total of 9 different mixtures. Additionally, reference trials with no PMHS were evaluated. Two factors, H and T, were also evaluated; therefore, the number of trials in these case are 2^2^ plus the central point, which would give us 5 different mixtures. In total, 14 different mixtures were evaluated, which were replicated 8 times each. Therefore, there were 112 preparations in total.

In order to better understand the reason for the selection of the levels considered for each factor in the investigation, we must consider information from the industrial manufacture. Plasterboard manufacturing includes stirring speeds of 350 rpm to 380 rpm, with agitation times in the mixer of 2–8 s and a convection drying stage with air temperatures decreasing from 315 °C to 178 °C [49]. To replicate an industrial mixer, the linear speed of the blades was equalized. The angular velocity by radius had to be equivalent to linear velocity to achieve an analogous model. To that end, an OSTER XPERT BLST3A-CPG052 blender with rotor radius of 2 cm to 3 cm was used, demanding an angular velocity of 10,000 rpm. Stirring speed increased 10–30 times to resemble real industrial conditions [50]. Industrial drying was replicated with a fan dryer to achieve forced convection drying, which ensures extraction of excess moisture without affecting the calcium sulfate dihydrate [51,52,53,54].

International standards were used to measure the impact of plasterboard preparation conditions on the properties of interest: water absorption (A, in percentage), flexural strength (Rf, in N), and thermal conductivity (Ct, in W/(m°K)). Water absorption and flexural strength percentages were measured according to the UNE-EN 520 standard [55]. Thermal conductivity was measured using the Hot Disk TPS 1500, compliant with the UNE-EN ISO 22007-2 standard [56].

**Figure 1:**
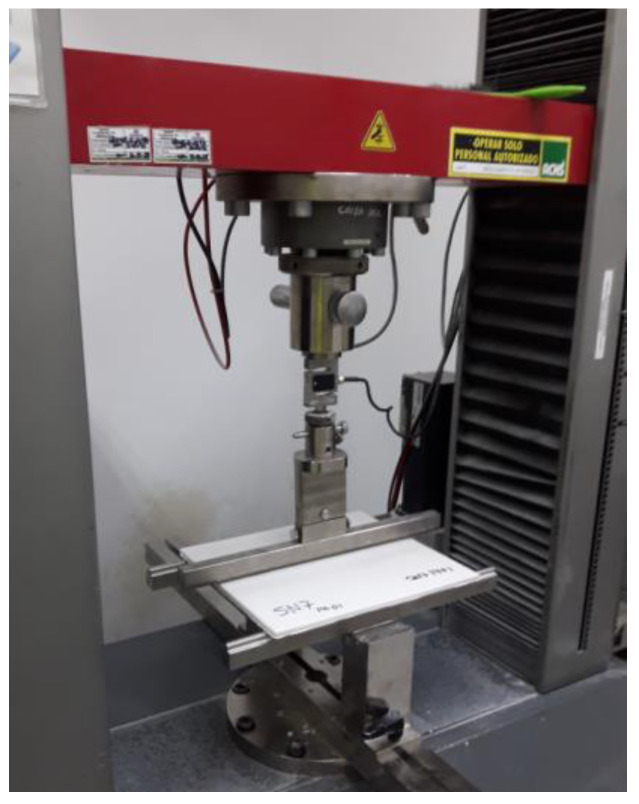
Equipment used to measure the flexural strength of plasterboard.

The flexural strength of gypsum boards must be characterized by its ultimate flexural load. The board must be subjected to a load that increases in a controlled manner until failure occurs. The equipment used is a loading device with an accuracy of 2%, capable of applying the necessary load at a speed of (250 ± 125) N/min. The board were placed on two parallel cylindrical supports with a diameter between 6 mm and 30 mm, and a distance between parallel axes of 350 mm. According to NCh 146/1 Of., the minimum value applied must be 110 N for a board with a thickness of 8 mm. Figure 1 shows an image of the equipment used to measure the flexural strength of plasterboard.

**Figure 2:**
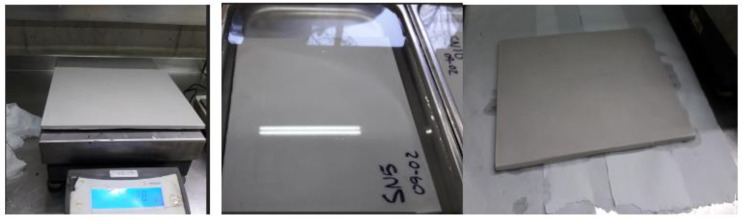
Board immersed in water for the water absorption test.

The water absorption test was performed as follows: immerse the board horizontally in a water bath, maintaining a height of 25 mm ± 1 mm of water above the upper surface of the test piece. Ribs in the bottom of the container are placed so that the bottom surface of the cylinder remains in contact with the water. The sample is kept submerged for two hours, and excess surface water is removed with blotting paper and weighed immediately, as shown in Figure 2. The increase in mass of the specimen corresponds to the water absorbed, and it is calculated as a percentage of the initial mass.

**Figure 3:**
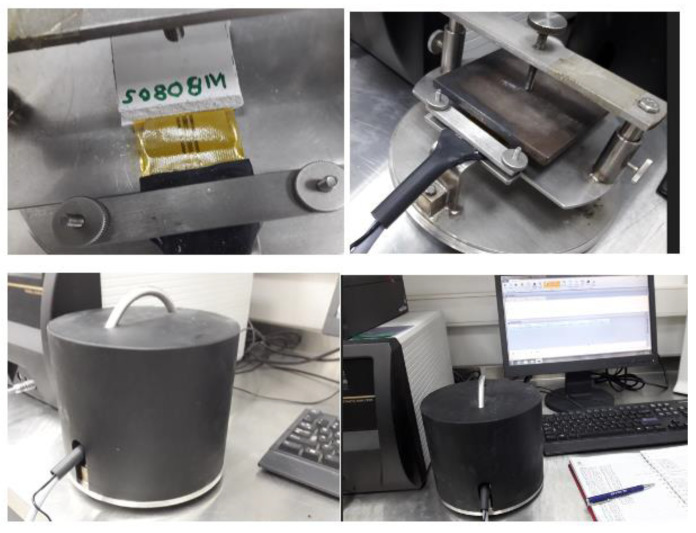
Hot Disk TPS 1500 equipment for the measurement of thermal conductivity.

Hot Disk TPS 1500 is the thermal conductivity meter for testing building materials, insulating materials, or any other type of large bulk samples. The TPS 1500 thermal conductivity range is 0.01 to 400 W/m/K, and the instrument handles temperatures from −100 °C to 750 °C. Samples can be up to a few millimeters thick. The TPS 1500 complies with ISO 22007-2 and carries the CE mark. A specimen containing an integrated hot disk sensor with negligible heat capacity is permitted to equilibrate at a given temperature. A heat spike in the form of a step function is produced by an electrical current through the sensor to generate a dynamic thermal field within the specimen. The increase in sensor temperature is measured as a function of time. Next, the response is analyzed according to the model developed for the specific sensor and assuming the environmental conditions. The sensor used was C5465 de 3.2 mm in radius. The sensor is a bifilar spiral made of etched metal foil, (10 ± 2) µm thick and covered on both sides by means of a thin insulating film (from 7 µm to 100 µm). Our samples were 40 mm × 40 mm with a thickness of 8 mm. Figure 3 shows the sequence of placing the sensor and the samples in the Hot Disk TPS 1500 equipment for the measurement of thermal conductivity.

To prepare the samples, a constant water/gypsum ratio of 0.80 was established for all trials, as suggested by Lootens et al. [50]. Water and PMHS, previously mixed manually, were added to the mixer container, and calcium sulfate hemihydrate was incorporated and stirred for the time indicated in the design matrix. Subsequently, the mixture was poured into silicone molds of 30 mm × 26 mm × 8 mm, unmolded after 30 min, and placed for 15 min in a fan oven at the temperatures indicated in the design matrix. The same oven was then left at 40 °C for 19 h and in room conditions (21 ± 2 °C and 51 ± 7%RH) until the time of testing.

### 2.3. Experimental Design

Table 1 shows trial details for each experiment in the experimental design matrix. Statistically, we want to establish whether the values obtained from a property are significantly different if we compare samples without and with PMHS. A null hypothesis is established that indicates that there really are no changes, and an alternative hypothesis that indicates that there is a change. The *p*-value is the probability that the null hypothesis is true or not. In general, the value of *p* considered is 0.05, which indicates that if the *p* calculated with the data gives us less than that value, then the null hypothesis is rejected and the alternative hypothesis is approved, which would indicate that there is a statistically significant difference.

Statistical software Minitab^®^ v19.1.1.0 (Minitab, LLC, State College, PA, USA) was used for design analysis and data processing.

## 3. Results and Discussion

### 3.1. Component Characterization

The X-ray fluorescence (XRF) characterization of the HH is shown in Table 2, indicating a chemical composition of 45% SO_3_ and 39.38% CaO. On the other hand, Table 3 shows the mineralogical composition measured with X-ray diffraction (XRD), indicating that the sample contained an 83.3% mass in weight in the form of calcium sulfate hemihydrate.

**Figure 4:**
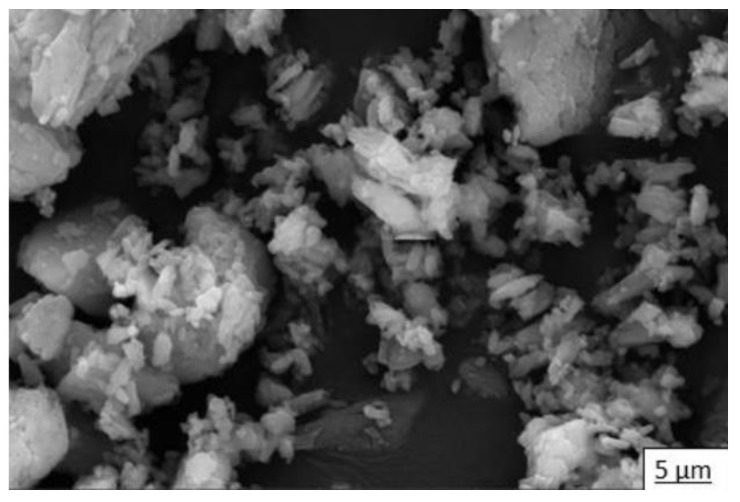
Microstructure of commercial calcium sulfate hemihydrate (HH).

**Figure 5:**
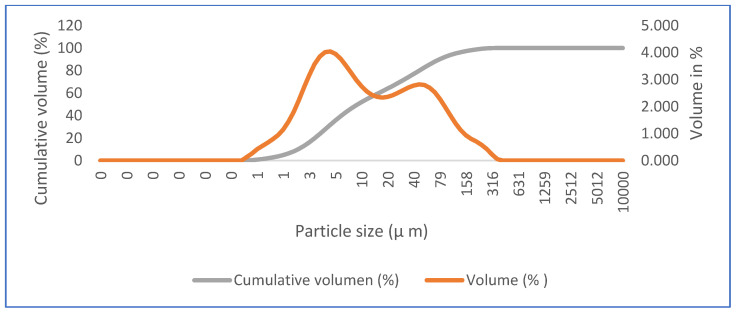
Particle size distribution of commercial calcium sulfate hemihydrate (HH).

In addition to the elemental and mineralogical characterization, complementary analyses of particle structure and size were performed via SEM and LDA, respectively. In Figure 4, we see the microstructure of commercial calcium sulfate hemihydrate (HH) by SEM, in which short crystals typical of bassanite and large crystals of calcite can be observed. Figure 5 shows the particle size distribution of HH. According to the laser scan diffraction analysis, the particle size distribution shows that 100% in volume of particles has less than 316 microns, 90% has less than 78.6 microns, 50% has less than nine microns, and 10% has less than two microns.

**Figure 6:**
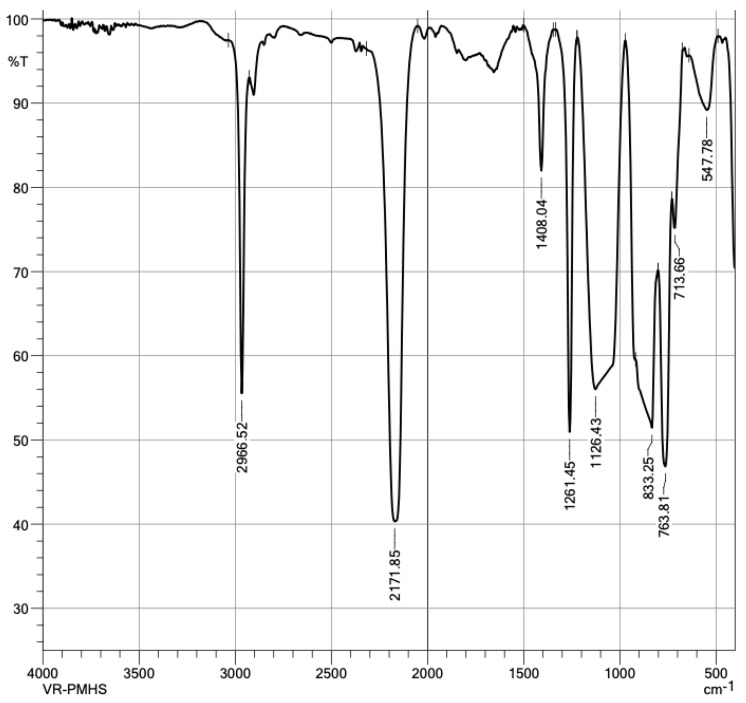
Infrared spectra of polymethylhydrosiloxane.

**Figure 7:**
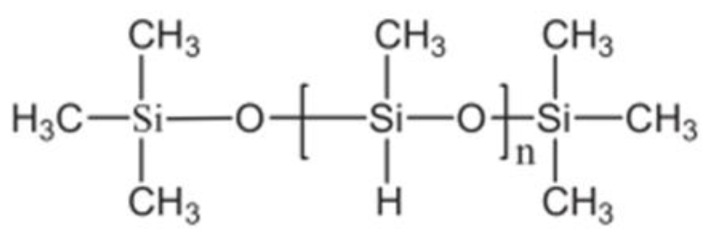
Polymethylhydrosiloxane.

Given the nature of a polymer, it is convenient to characterize PMHS with an FTIR analysis of the sample. The spectrum presents apodization with Happ–Genzel (The Happ–Genzel function is often used to achieve a good balance between ripple size and resolution). Figure 6 identifies PMHS functional groups. As expected, the vibration signal at 2.966 cm^−1^ was asymmetric by the stretching of C–H bonds of group CH_3_, the signal at 2.171 cm^−1^ corresponded to Si-H stretching, the 1.408 cm^−1^ signal to asymmetric bending of the Si-CH_3_ bond, at 1.261 cm^−1^ to Si-CH_3_ symmetrical bending vibration, at 1.126 cm^−1^ to asymmetric Si-O-Si stretching, at 833 cm^−1^ to scissor-type bending of the Si-H bond, and at 763 cm^−1^ to the vibration of Si-C stretching [29]. Figure 7 identified the groups matching those expected in the PMHS structure [35].

XRD measurements were made of one DH sample without PMHS and another DH sample with 0.2% de PMHS. When comparing the results, we see that the hydration reaction has occurred almost completely because in the XRD the presence of HH is not observed, and only DH and the calcite has not been affected. Anhydrite does not show up in the XRD results because it is amorphous, but the FTIR results of the samples are able to detect the presence of anhydrite as it is shown later. 

### 3.2. Results Analysis

#### 3.2.1. Experiment Results

Summaries of the results of all trials performed are shown in Table 4 and Table 5. Table 4 shows the average of the eight replications and standard deviation for the measured value of water absorption (A), flexural strength (Rf), and thermal conductivity (Ct). Table 5 shows the porosity and length-to-width ratio for a selected group of samples.

As Table 4 shows, the average water absorption of trials without PMHS was 48%, and with PMHS it was 3.5%, a significant reduction of 93%. On the other hand, the average flexure strength of trials without PMHS was 115 N, and with PMHS it was 114 N, a reduction of only 0.9%. Finally, the average thermal conductivity of trials without PMHS was 0.338 W/m°K, and with PMHS it was 0.320 W/m°K, a reduction of 5%. The data suggest that the polymer has a significant effect on water absorption, no effect on flexure strength, and only a slight effect on thermal conductivity.

From Table 4, by comparison, only the samples with PMHS show that water absorption is also reduced by increases in the drying temperature. In fact, water absorption (A) tends to decrease in most cases when temperature increases for a constant dosage and constant stirring time. However, at a high enough drying temperature, water absorption hardly changes, suggesting a limit for the effect. In general, it could be said that for a sample with PMHS the lowest values of water absorption are achieved with the highest values of stirring time and drying temperature, but there is a point after which no further water absorption reduction can be achieved.

Given the results shown in Table 4, a subgroup of tests was selected for porosity and crystal morphology analysis. The subgroup selected for the trials without PMHS at the minimum, medium, and maximum levels of agitation and temperature corresponded to trials SN1, SN5, and SN4. Likewise, a subgroup—N1, N9 and N8—of samples with PMHS was selected with the same purpose. The porosity and morphology results measured via X-ray micro-tomography (XMT) and SEM are shown in Table 5. Samples with added PMHS showed significantly higher porosity than those without added PMHS, and crystals formed with the addition of PMHS were more compact.

From Table 5 it can be observed that when going from low dosage, low stirring time, and low drying temperature (SN1) to high conditions in dosage, high stirring time, and high temperature (N8), the length-to-width ratio (L/W) of the crystals decreases and porosity increases. In addition, a weak reduction in L/W is observed when the temperature increases or the stirring time increases. Likewise, it can be observed that there is a consistent increment of porosity dosage with PMHS dosage increases, which may be due to the generation of more trapped air bubbles. No relationship between porosity and L/W by itself can be attributed to stirring time or drying temperature because additional experiments should be run where the two other variables are kept constant. In general, we can conclude that the incorporation of PMHS causes significant changes in the morphology and porosity of the gypsum boards, and variations in the operational manufacturing conditions can contribute to additional improvements due to changes in L/W ratio. In fact, morphology changes are observed when all samples without PMHS are compared with samples with PMHS at constant stirring time and drying temperature. In the case of SN1 as compared to N1, we observe a decrease from 3.38 to 2.93 in L/W ratio, a −13.31% reduction. In the case of SN5 as compared to N9, a decrease in L/W ratio of 28.95% is observed. In the case of SN4 as compared to N8, a decrease in L/W ratio of 30.96% is observed. As L/W decreases, porosity increases as well. In the case of SN1 as compared to N1, the porosity increases from 0.513% to 1.494, an increase in 191.23%. An increase in porosity of 322.63% and 580.14 is observed when SN5 vs. N9 and SN4 vs. N8 are compared, respectively.

#### 3.2.2. Crystal Morphology

**Figure 8:**
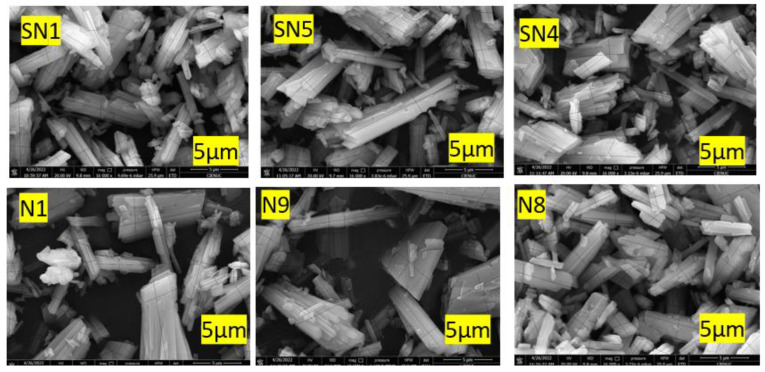
(SN1) without PMHS minimum level, (SN5) medium level, (SN4) maximum level, (N1) with PMHS minimum level, (N9) medium level, (N8) maximum level.

The trials were conducted according to the experimental matrix in Table 1. Morphology results for samples without PMHS addition (SN1, SN5, SN4) and PMHS addition (N1, N9, N8) are shown in Figure 8. The figures were analyzed with IMAGEJ software to determine average crystal length and width. Averages of 50 crystals are indicated in Table 5.

A Minitab data analysis evidenced a strong relationship between PMHS addition, agitation, and drying temperature in morphology as related to L/W. The results of the hypothesis paired t-test show a *p* value below 0.05 for N9 and N8, corresponding to the factors at medium and maximum level compared to the equivalent trials without the addition of PMHS, SN5, and SN4. In these cases, added PMHS made a significant difference in morphology by reducing the particle L/W ratio. However, at the minimum level, meaning low temperature and low stirring time, the test could not determine an impact on morphology after the addition of PMHS. The results suggest there is a minimum temperature and stirring that need to be provided to affect the crystal morphology.

To define the effect of each factor (D, H, and T) on the morphology of the DH formed, the following analysis was conducted:

1. Only the stirring time (H) effect was evaluated by mixing for 20 s, 40 s, and 60 s. The reactions were stopped by adding isopropyl alcohol, washing, and drying. The DH powder obtained was analyzed by SEM, showing a length–width ratio of 50 crystals. Although it is observed that the average of this ratio decreases when stirring time increases, statistically it does not present a significant difference.
2. The stirring time and drying temperature synergetic effect over the length–width ratio was also evaluated. A significant difference at medium and maximum levels in morphology with respect to the reference was observed.
3. The effect of the length–width ratio was finally evaluated when all three factors (D, H, T) were changed simultaneously. In this case, a significant effect on the morphology between without and with PMHS addition at the medium and maximum levels of stirring time and temperature was observed.

Summarizing, the addition of PMHS causes a statistically significant difference in morphology between trials without and with PMHS addition, at the medium and maximum levels of stirring time and temperature. Moreover, the higher the PMHS dosage and temperature, the lower is the length–width ratio of crystals.

#### 3.2.3. Porosity

**Figure 9:**
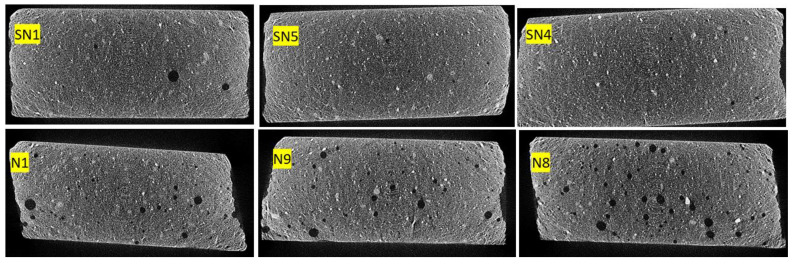
Porosity (in two dimensions) without and with PMHS at minimum, medium, and maximum levels of factors.

**Figure 10:**
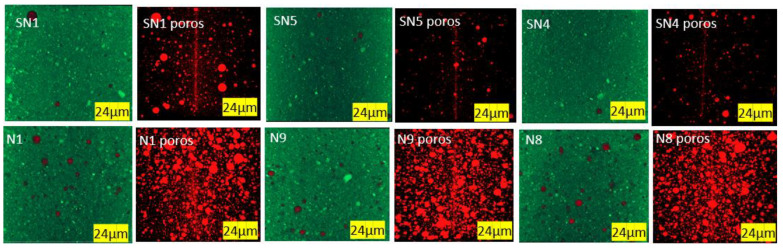
Porosity (in 3D) without and with PMHS at minimum, medium, and maximum levels of factors. Air pores are identified in red.

Porosity was measured by analyzing 3D images obtained via X-ray microtomography. Figure 9 shows samples, with scattered grains of calcite in white and dispersed air pores in black. Figure 10 shows the same samples where red air pores are identified via image analysis. Image segmentation makes it possible to assess the porosity level quantitatively.

As indicated in Table 5 and observed in Figure 6 and Figure 7, the total porosity (Po, in vol%) obtained by means of the X-ray microtomography technique shows that the incorporation of PMHS causes a considerable generation of trapped air bubbles, which reached maximum values when PMHS dosage, agitation, and drying temperature increased.

**Figure 11:**
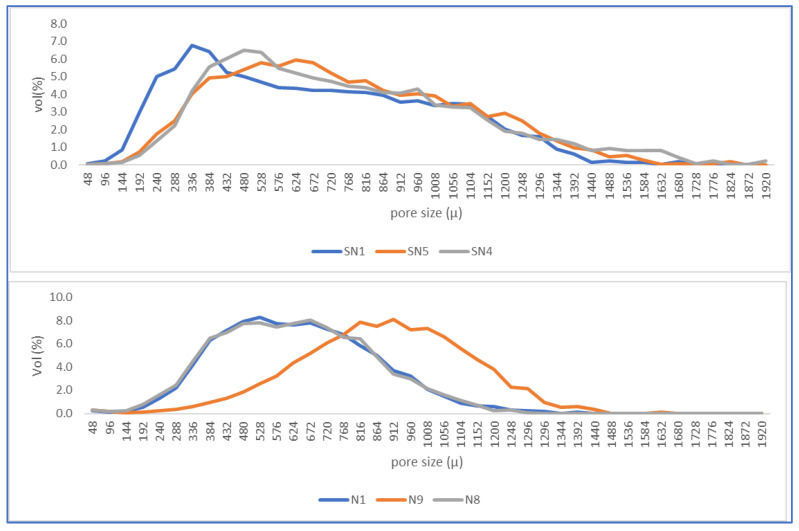
Pore size distribution without (above) and with PMHS.

Figure 11 shows the distribution of pore sizes identified in the images. The upper figure shows the distribution for samples without PMHS and the lower figure with PMHS addition. It can be clearly observed that there is a shift of the average pore size to the right, showing how PMHS addition increased the size of trapped pores with respect to DH without PMHS. There seems to be an optimal dose that maximized the number of pores in the DH matrix because the size distribution was clearly coarser in the N9 conditions with medium levels: 0.6% dosage, 40 s of stirring time, and a drying temperature of 140 °C.

Minitab analysis was used to determine if there was a significant difference in trapped air pores between trials. The results of the statistical analysis (Paired *t*-test—Minitab) showed that because *p* is below 0.05, there is a significant difference in the number of pores because of the addition of PMHS.

**Figure 12:**
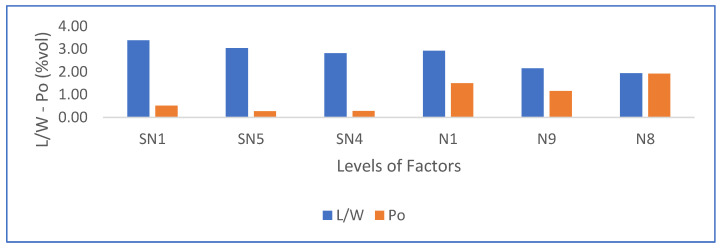
Effects on length–width ratio and porosity due to the increment in PMHS dosage, stirring time, and drying temperature according to Table 5.

Figure 12 shows a summary of the results. As shown, porosity increases with increments of agitation time, drying temperature, and PMHS doses, while at the same time length-to-width ratio decreases.

#### 3.2.4. Water Absorption (%)

As indicated in Table 4, the addition of PMHS significantly decreased the water absorption rate, which was observed previously by Issa and Luyt [37], who indicated that PMHS hydrophilic groups bind to hydrophilic groups on the HH surface after hydrolysis and fill the pores between the crystals. Additionally, our results showed that the incorporation of PMHS increased the amount of trapped air and porosity, while decreasing crystal length–width ratio. The reduction in water absorption may be due to the hydrophobicity of PMHS that covers the micropores, repelling water and preventing water in the macropores from infiltrating the matrix. This phenomena has also been observed in a different application [25].

**Figure 13:**
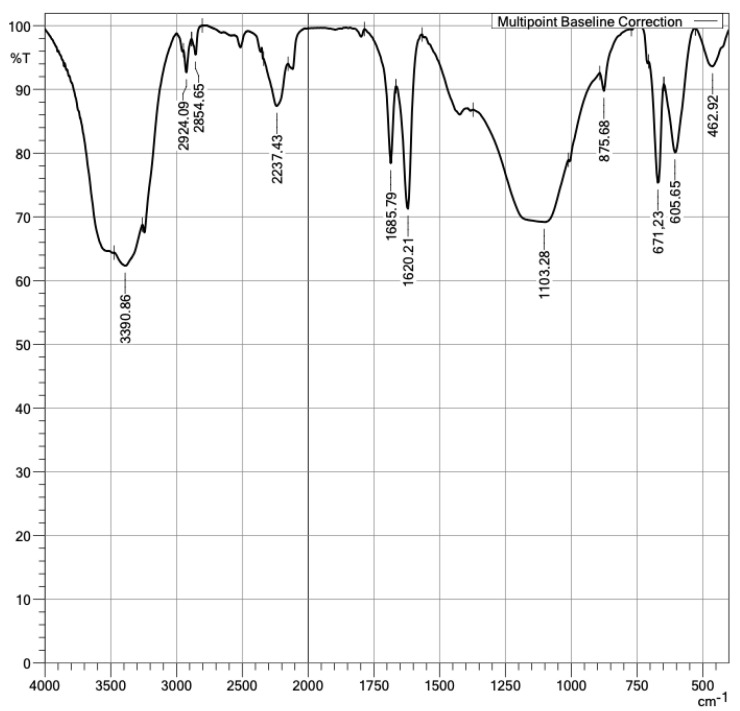
Infrared spectra of DH without PMHS.

**Figure 14:**
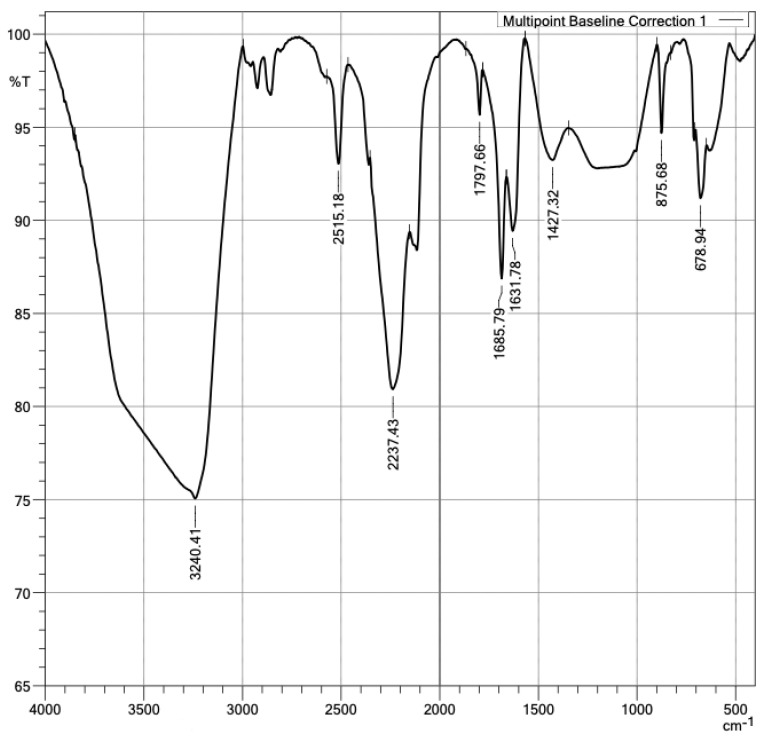
Infrared spectra of DH with PMHS.

In addition, the presence of PMHS functional groups in the samples’ surfaces was confirmed. With the FTIR technique, the functional groups in a plasterboard without and with PMHS were measured, as shown in Figure 13 and Figure 14. The difference in spectrum between the sample without PMHS in Figure 13 versus the sample with PMHS shown in Figure 14 can be clearly observed. In Figure 13, the signal at 1.103 cm^−1^ identified asymmetric stretch vibration and symmetric bending vibrations at 462 cm^−1^ and asymmetrical ones at 605 cm^−1^ and 671 cm^−1^ of the sulfate group (SO_4_^−^), indicating the presence of dihydrate. Sulfate as anhydrite is distinguished from sulfate as gypsum by an absorption band at 2.237 cm^−1^. As expected, water vibrations were observed at 3.390 cm^−1^, and the two water bending vibrations were observed at 1.620 cm^−1^ and 1.685 cm^−1^. The presence of carbonate was confirmed by the signal at 875 cm^−1^ [57,58,59]. In contrast, Figure 14 shows a signal at 1.427 cm^−1^ due to the Si-C asymmetric bending vibration of SiCH_3_. A signal between 2900 cm^−1^ and 2.980 cm^−1^ was also observed, associated with the C–H stretching of CH_3_. Carbonate was observed in the signals at 875 cm^−1^, 2.515 cm^−1^, and 1.797 cm^−1^ [60]. The FTIR technique also allowed identifying anhydrite sulfate in the signals at 2.237 cm^−1^ and 678 cm^−1^ of the dihydrate. As expected, water vibrations were observed at 3.240 cm^−1^ and water bending vibrations at 1.685 cm^−1^ and 1.631 cm^−1^. In addition, a signal was observed at 1.427 cm^−1^, associated with the Si-C vibration of SiCH_3_. Finally, between 1.250 cm^−1^ and 1.050 cm^−1^, the vibration of Si-O-Si was observed [59,61,62]. The presence in the surface of PMHS functional groups promotes hydrophobic forces repelling water, and therefore it may contribute to the reduction of water absorption.

**Figure 15:**
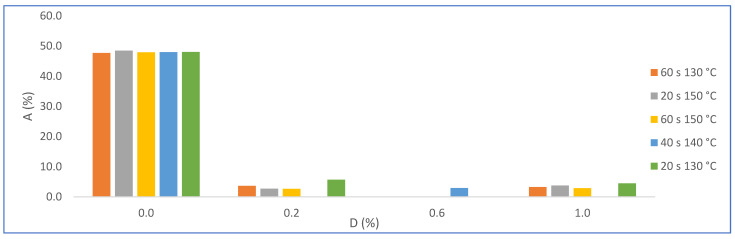
Effects on water absorption (A) for factor groups by increasing PMHS dosage (from 0.0% to 1.0%.), stirring time, and drying temperature.

**Figure 16:**
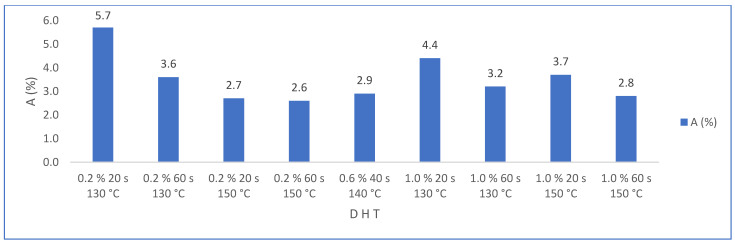
Effects on water absorption (A) for factor groups by increasing PMHS dosage (from 0.2% to 1.0%.), stirring time, and drying temperature.

The statistical evaluation of water absorption results (Hypothesis test—2-sample *t*-test—Minitab) show a significant difference because the *p* value was less than 0.05. Figure 15 shows the results of water absorption (A) by varying the amount of PMHS dosage (D) for groups of samples with the same stirring time (H) and drying temperature (T). It is observed that water absorption decreases drastically when incorporating the PMHS, from an average of 48% to 3.5% with PMHS, a variation of 93%. In contrast, it is not observed that water absorption changes significatively when the dosage increases from 0.2% to 1.0% of PMHS for any level of H and T. However, Figure 16, shows that the lowest absorption value is obtained with a PHS dosage between 0.2% and 0.6%, with drying temperature between 140 °C and 150 °C for any value of stirring time. Chen et al. [41] observed a similar behavior with different polymers, attributing that a high polymer concentration inhibits the growth of the crystals, causing a decrease in crystallinity and, although the length–width ratio is reduced, there would be the presence of more loose structures between the crystals.

#### 3.2.5. Flexural Strength (N)

**Figure 17:**
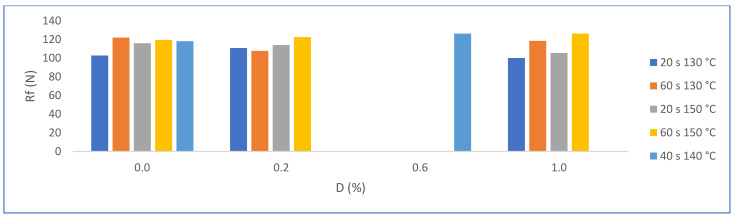
Effects on Rf for factor groups by increasing PMHS dosage (from 0.0% to 1.0%.), stirring time, and drying temperature.

It was expected that the formation of more compact crystals would favor the flexural strength, but the increase in the size of the trapped pores leads to a nullifying effect. In fact, the statistical evaluation indicates that there is no significant difference between the results obtained from trials without and from those with PMHS; see Figure 17.

#### 3.2.6. Thermal Conductivity (W/(m°K))

**Figure 18:**
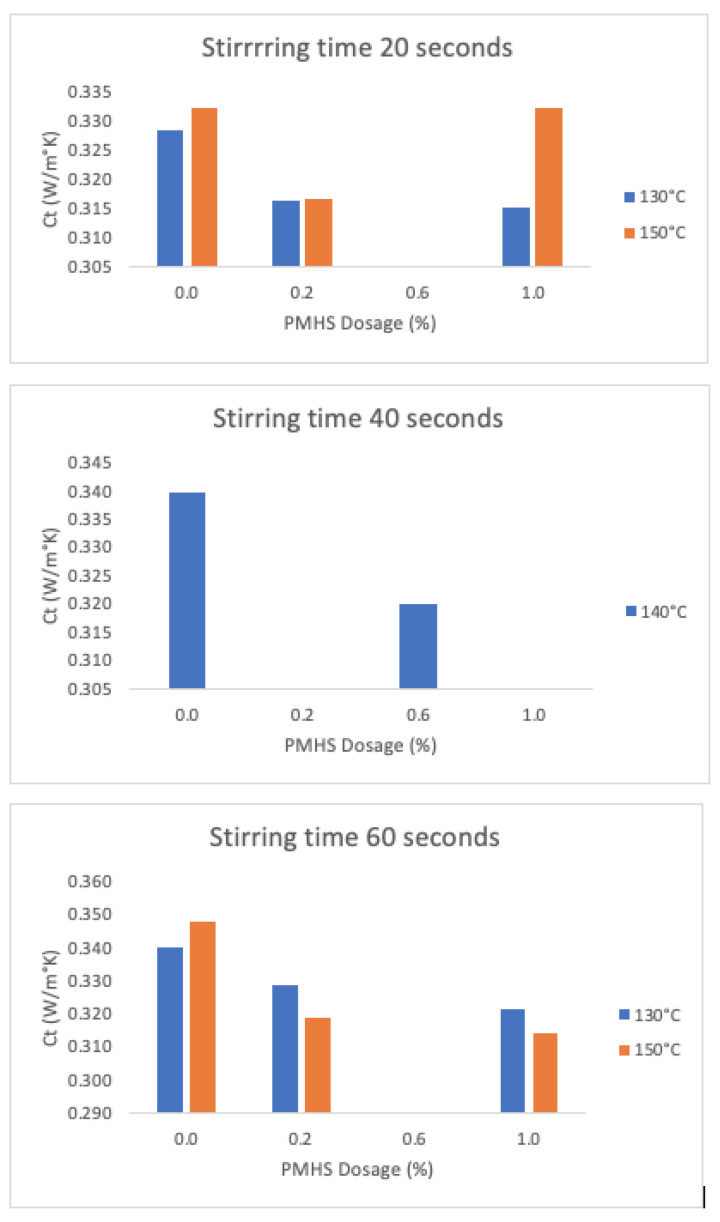
Effects on thermal conductivity as a function of PMHS dosage (from 0.0% to 1.0%.), stirring time, and drying temperature.

Figure 18 shows the results for trials with constant stirring time (H) and drying temperature (T) values as functions of PMHS dosage. The greatest variation in thermal conductivity between without and with PMHS is a reduction of 10%, which is achieved at high H and high T level conditions (trial SN4 as compared to N8), and a reduction of 4% in the low H and low T level conditions (trial SN1 as compared to N1).

Statistical indicator *p* for comparison between the average conductivity for samples with and without PMHS dosage is below 0.05, indicating that there is a significant difference between them. The higher the porosity, the lower the value of thermal conductivity, because the air trapped in the DH structure is less conductive of the heat flow.

As indicated in Section 3.2.3, the incorporation of PMHS increased the size of the trapped pores with respect to DH without PMHS. There seems to be an optimal dose that maximizes the variation of the pore size in the DH matrix. The pore size mode increases from 528 μm to 912 μm, a 73% increase, when the sample was prepared at 0.6% PMHS dosage, 40 s, and 140 °C (trial N9) as compared with a sample with no PMHS dosage (trial SN5). At the low-level conditions (SN1 compared to N1), the pore size mode increase was 57%, from 336 microns to 528 microns. At the high-level conditions (SN4 compared to N8), the pore size mode increase was 40%, from 480 to 672 microns. In all cases, the addition of PMHS increased the porosity and the pore size.

**Figure 19:**
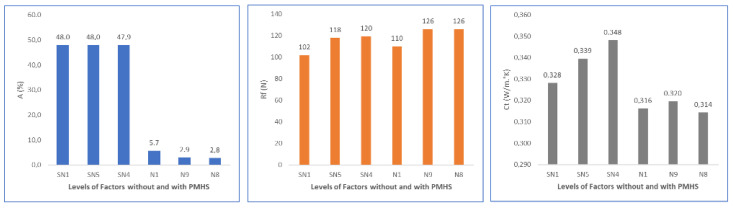
Effects on water absorption, flexural strength, and thermal conductivity as functions of PMHS dosage, stirring time, and drying temperature.

Finally, Figure 19 shows a summary for water absorption (A), flexural strength (Rf), and thermal conductivity (Ct) in function of D, H, and T. Water absorption decreased significantly with the addition of PMHS, increased agitation, and increased drying temperature. Flexural strength was not affected. Thermal conductivity decreased as PMHS dosage, agitation, and drying temperature increased.

## 4. Conclusions

This paper analyzed the hygrothermal and mechanical performance of plasterboards as produced by mixing water and calcium sulfate hemihydrate, as functions of the polymethylhydrosiloxane dosage, variation of the stirring or homogenization time, and final drying temperature. The effects on morphology and porosity and how they affect the water absorption, flexural strength, and thermal conductivity of plasterboard were evaluated.

The data show that adding PMHS to the hydration reaction of calcium sulfate hemihydrate has a significant impact on water absorption, and it also has some lower effect on thermal conductivity. By variations in PMHS dosage, mixing time, and the temperature at which the gypsum boards are dried, the setting morphology of the final particles can be significantly affected. As these variables increase, crystal length–width ratio tended to decrease and total porosity increased. As a result, the water absorption and thermal conductivity decreased as well. The experimental data suggest that most of the impact is due to the PMHS dosage. Once a PMHS dosage is applied, the data also suggest there is a maximum dosage for a given stirring time and drying temperature that maximizes improvement at a minimum effort. The analysis of the data shows no effect on the flexural strength of the plasterboard.

The data show an optimum dosage of PMHS, over which the positive effect in the reduction of water absorption and the reduction in thermal conductivity is nullified. There is evidence that the optimal value would possibly be around 0.6% PMHS dosage in weight for the levels under evaluation.

The results obtained in this investigation contribute to the knowledge of the relation between polymer PMHS addition, stirring time, and drying temperature as driving factors that affect crystal morphology and porosity in the plasterboard, and therefore in its performance in water absorption and thermal conductivity.

However, there are still questions to solve in order to improve the flexural strength performance of the plasterboard without negative interference with the other two properties, water absorption and thermal conductivity. We are conducting additional research to study the improvement of the plasterboard flexibility by the addition of complementary elements mixed under controlled polymethylhydrosiloxane dosage, stirring, and drying temperature. Plastic micro-waste, such as that resulting from the mechanical recycling of polyethylene terephthalate, could fulfill this function.

## Figures and Tables

**Table 1:** Experimental design matrix.

	Trial	D (%)	H (s)	T (°C)	Water/HH	Number of Replicates
Trials without PMHS	SN1	0.0	20	130	0.8	8
	SN2	0.0	60	130	0.8	8
	SN3	0.0	20	150	0.8	8
	SN4	0.0	60	150	0.8	8
	SN5	0.0	40	140	0.8	8
Trials with PMHS	N1	0.2	20	130	0.8	8
	N2	1.0	20	130	0.8	8
	N3	0.2	60	130	0.8	8
	N4	1.0	60	130	0.8	8
	N5	0.2	20	150	0.8	8
	N6	1.0	20	150	0.8	8
	N7	0.2	60	150	0.8	8
	N8	1.0	60	150	0.8	8
	N9	0.6	40	140	0.8	8

**Table 2:** Chemical composition of calcium sulfate hemihydrate used in this experiment (wt%).

SO_3_	CaO	SiO_2_	Al_2_O_3_	Fe_2_O_3_	SrO	K	LOI
45.00	39.83	1.10	0.50	0.18	0.17	0.05	13.18

**Table 3:** Mineralogical composition of calcium sulfate hemihydrate.

Name	Formula	Presence (wt%)
Bassanite	CaSO_4_·0.5H_2_O	83.2
Calcite	CaCO_3_	10.8
Anhydrite	CaSO_4_	6.0

**Table 4:** Results of the A, Rf, and Ct trials performed according to the experimental design matrix.

Matrix without PMHS	Trial	D (%)	H (s)	T (°C)	A (%)		Rf (N)		Ct (W/m°K)	
					Avg.	Dev.	Avg.	Dev.	Avg.	Dev.
	SN1	0.0	20	130	48.0	0.6	102	26	0.328	0.018
	SN2	0.0	60	130	47.7	0.6	122	28	0.340	0.012
	SN3	0.0	20	150	48.5	0.7	116	24	0.332	0.020
	SN4	0.0	60	150	47.9	0.6	120	40	0.348	0.008
	SN5	0.0	40	140	48.0	0.5	118	21	0.339	0.013
Matrix with PMHS	Trial	D (%)	H (s)	T (°C)	A (%)		Rf (N)		Ct (W/m°K)	
					Avg.	Dev.	Avg.	Dev.	Avg.	Dev.
	N1	0.2	20	130	5.7	1.6	110	15	0.316	0.015
	N2	1.0	20	130	4.4	3.5	100	18	0.315	0.010
	N3	0.2	60	130	3.6	2.2	108	16	0.329	0.005
	N4	1.0	60	130	3.2	1.1	118	27	0.322	0.011
	N5	0.2	20	150	2.7	1.1	114	24	0.316	0.016
	N6	1.0	20	150	3.7	2.2	105	6	0.332	0.037
	N7	0.2	60	150	2.6	1.2	123	26	0.319	0.018
	N8	1.0	60	150	2.8	1.7	126	18	0.314	0.028
	N9	0.6	40	140	2.9	1.9	126	26	0.320	0.012

**Table 5:** Results of the length-to-width ratio (L/W) and porosity (Po) tests performed according to the experimental design matrix.

Trial	D (%)	H (s)	T (°C)	L/W		Po (%vol)
				Avg.	Dev.	
SN1	0.0	20	130	3.38	1.93	0.513
SN5	0.0	40	140	3.04	2.10	0.274
SN4	0.0	60	150	2.81	1.48	0.282
N1	0.2	20	130	2.93	1.30	1.494
N9	0.6	40	140	2.16	1.30	1.158
N8	1.0	60	150	1.94	1.01	1.918

## References

[1] Singh N., Middendorf B. (2007). Calcium sulphate hemihydrate hydration leading to gypsum crystallization. Prog. Cryst. Growth Charact. Mater..

[2] Tinkova T., Grigorova I., Nishkov I. (2016). New Approaches on Gypsum Body Composite Materials Addition. Researchgate.

[3] Chen X., Wu Q., Gao J., Tang Y. (2021). Hydration characteristics and mechanism analysis of β-calcium sulfate hemihydrate. Constr. Build. Mater..

[4] Yu Q., Brouwers H. (2011). Microstructure and mechanical properties of β-hemihydrate produced gypsum: An insight from its hydration process. Constr. Build. Mater..

[5] Polat S., Sayan P. (2019). Ultrasonic Irradiation during the Calcium Sulfate Hemihydrate to Dihydrate Transformation Process. Chem. Eng. Technol..

[6] de Korte A.C.J., Brouwers H.J.H. (2011). Ultrasonic sound speed analysis of hydrating calcium sulphate hemihydrate. J. Mater. Sci..

[7] De Korte A.C.J., Brouwers H.J.H. Ultrasonic sound speed measurement as method for the determining the hydration degree of gypsum. Proceedings of the 8th FIB International PhD Symposium in Civil Engineering.

[8] Adrien J., Meille S., Tadier S., Maire E., Sasaki L. (2016). In-situ X-ray tomographic monitoring of gypsum plaster setting. Cem. Concr. Res..

[9] Kamalipour M., Dehghani S.A.M., Naseri A., Abbasi S. (2017). Role of agitation and temperature on calcium sulfate crystallization in water injection process. J. Pet. Sci. Eng..

[10] Li J.Q., Gong M.Z., Li G.Z. (2012). Relationship between Exothermic Effect and Crystal Growth in the Hydration Process of Waterproof Gypsum. Appl. Mech. Mater..

[11] Roveri M., Goidanich S., Dotelli G., Toniolo L. (2020). Semi-empirical models to describe the absorption of liquid water in natural stones employed in built heritage before and after the application of water repellent treatments. Constr. Build. Mater..

[12] Nicoleau L., Van Driessche A.E., Kellermeier M. (2019). A kinetic analysis of the role of polymers in mineral nucleation. The example of gypsum. Cem. Concr. Res..

[13] Aquilano D., Otálora F., Pastero L., García-Ruiz J.M. (2016). Three study cases of growth morphology in minerals: Halite, calcite and gypsum. Prog. Cryst. Growth Charact. Mater..

[14] Mróz P., Mucha M. (2018). Hydroxyethyl methyl cellulose as a modifier of gypsum properties. J. Therm. Anal. Calorim..

[15] Czaderna A., Kocemba A., Kozanecki M., Mucha M., Mróz P. (2018). The influence of cellulose derivatives on water structure in gypsum. Constr. Build. Mater..

[16] Moghadam H.A., Mirzaei A. (2019). Comparing the effects of a retarder and accelerator on properties of gypsum building plaster. J. Build. Eng..

[17] Polat S., Sayan P. (2018). Assessment of propionic acid adsorption performance on the phase transformation of calcium sulfate hemihydrate to dihydrate. Sep. Sci. Technol..

[18] Mucha M., Mróz P., Wrona D. (2017). Chitosan applied for gypsum modification. Prog. Chem. Appl. Chitin Deriv..

[19] Mucha M., Mróz P., Kocemba A. (2016). Polymer composites based on gypsum matrix. AIP Conf. Proc..

[20] Garg M., Pundir A., Singh R. (2015). Modifications in water resistance and engineering properties of β-calcium sulphate hemihydrate plaster-superplasticizer blends. Mater. Struct..

[21] Zhou P.-P., Wu H.-C., Xia Y.-M. (2016). Influence of synthetic polymers on the mechanical properties of hardened β-calcium sulfate hemihydrate plasters. J. Ind. Eng. Chem..

[22] Huang X., Li C., Zuo K., Li Q. (2020). Predominant Effect of Material Surface Hydrophobicity on Gypsum Scale Formation. Environ. Sci. Technol..

[23] Mróz P., Mucha M. (2017). Hydration kinetics of calcium sulphate hemihydrate modified by water-soluble polymers. Int. J. Eng. Res. Sci..

[24] Li J., Li G., Yu Y. (2007). The influences of gypsum water-proofing additive on gypsum crystal growth. Mater. Lett..

[25] Pan H., Li G. (2013). Emulsion Waterproof Agent and Its Effects on Intrinsic Properties of Gypsum. Asian J. Chem..

[26] Wang Q., Cui Y., Xue J. (2019). Study on the improvement of the waterproof and mechanical properties of hemihydrate phosphogypsum-based foam insulation materials. Constr. Build. Mater..

[27] Kondratieva N., Barre M., Goutenoire F., Sanytsky M. (2017). Study of modified gypsum binder. Constr. Build. Mater..

[28] Ding Y., Fang Y., Ren Q., Fang H., Zhang Q., Oh W.C. (2015). Study on the Waterproofing Performance of FGD Gypsum Building Products from Inorganic-Organic Composite Additives. Korean J. Mater. Res..

[29] Li Z., Xu K., Peng J., Wang J., Zhang J., Li Q. (2021). Study on mechanical strength and water resistance of organosilicon waterproofing agent blended recycled gypsum plaster. Case Stud. Constr. Mater..

[30] Wu Q., Ma H., Chen Q., Gu B., Li S., Zhu H. (2019). Effect of silane modified styrene-acrylic emulsion on the waterproof properties of flue gas desulfurization gypsum. Constr. Build. Mater..

[31] Heim D., Mrowiec A., Pralat K., Mucha M. (2018). Influence of Tylose MH1000 Content on Gypsum Thermal Conductivity. J. Mater. Civ. Eng..

[32] Rahmanian I., Wang Y. (2012). A combined experimental and numerical method for extracting temperature-dependent thermal conductivity of gypsum boards. Constr. Build. Mater..

[33] Kondratieva N., Barre M., Goutenoire F., Sanytsky M., Rousseau A. (2020). Effect of additives SiC on the hydration and the crystallization processes of gypsum. Constr. Build. Mater..

[34] Capasso I., Pappalardo L., Romano R.A., Iucolano F. (2021). Foamed gypsum for multipurpose applications in building. Constr. Build. Mater..

[35] Tönjes J., Longwitz L., Werner T. (2021). Poly(methylhydrosiloxane) as a reductant in the catalytic base-free Wittig reaction. Green Chem..

[36] Nagappan S., Choi M.-C., Sung G., Park S.S., Moorthy M.S., Chu S.-W., Lee W.-K., Ha C.-S. (2013). Highly transparent, hydrophobic fluorinated polymethylsiloxane/silica organic-inorganic hybrids for anti-stain coating. Macromol. Res..

[37] Issa A.A., Luyt A.S. (2019). Kinetics of Alkoxysilanes and Organoalkoxysilanes Polymerization: A Review. Polymers.

[38] Keller W. (1999). The Waterproofing of Gypsum with Organosilicon Compounds.

[39] Su D., Huang C., Hu Y., Jiang Q., Zhang L., Zhu Y. (2011). Preparation of superhydrophobic surface with a novel sol–gel system. Appl. Surf. Sci..

[40] Jakobsmeier L. (2000). Reaktivitat und Wechselwirkungen Siliciumorganischer Verbindungen in Einer CaSO_4_·2H_2_O—Matrix.

[41] Chen C., Ma F., He T., Kang Z., Wang Y., Shi C. (2022). Improved water and efflorescence resistance of flue gas desulfurization gypsum-based composites by generating hydrophobic coatings. J. Clean. Prod..

[42] Hajakian P., Reed P.W. (2017). Water-Resistant Gypsum Products and Methods. U.S. Patent.

[43] Hajakian P., Reed P.W. (2017). Water-Resistant Gypsum Products and Methods.

[44] Tilford R. (2018). Base-Mediated Hydrophobing Compositions and Processes. U.S. Patent.

[45] Instituto Nacional de Normalización-INN-Chile (1999). NCh 143. of 1999 Yeso Calcinado-Requisitos.

[46] Lin C.L., Videla A.R., Yu Q., Miller J.D. (2010). Characterization and analysis of Porous, Brittle solid structures by X-ray micro computed tomography. JOM.

[47] Videla A., Lin C., Miller J. (2008). Simulation of saturated fluid flow in packed particle beds—The lattice-Boltzmann method for the calculation of permeability from XMT images. J. Chin. Inst. Chem. Eng..

[48] Videla A., Lin C., Miller J. (2006). 3D characterization of individual multiphase particles in packed particle beds by X-ray microtomography (XMT). Int. J. Miner. Process..

[49] Kuntze R.A. (2009). Gypsum: Connecting Science and Technology.

[50] Lootens D., Ampudia M., Hampel C., Mueller M. (2012). Laboratory scaling of gypsum board production. ZKG Int..

[51] Seck M.D., Van Landeghem M., Faure P., Rodts S., Combes R., Cavalié P., Keita E., Coussot P. (2015). The mechanisms of plaster drying. J. Mater. Sci..

[52] Seck M.D., Keita E., Coussot P. (2018). Some Observations on the Impact of a Low-Solubility Ionic Solution on Drying Characteristics of a Model Porous Medium. Transp. Porous Media.

[53] Seck M.D., Keita E., Faure P., Cavalié P., Van Landeghem M., Rodts S., Coussot P. (2016). Subflorescence and plaster drying dynamics. Chem. Eng. Sci..

[54] Mohammed W., Alabidalkreem O., Hallosh A. (2022). Calculating the Effective Moisture Diffusivity during Drying Process of Gypsum Board. Int. Res. J. Innov. Eng. Technol..

[55] (2010). Gypsum plasterboards—Definitions, requirements and test methods.

[56] (2016). AENOR (Asociacion Española de Normalizacion y Certificacion), Plásticos-Determinación de la Conductividad Térmica y la Difusividad Térmica-Parte 2: Método de la Fuente de Calor Transitoria (Disco Caliente).

[57] Anbalagan G., Mukundakumari S., Murugesan K.S., Gunasekaran S. (2009). Infrared, optical absorption, and EPR spectroscopic studies on natural gypsum. Vib. Spectrosc..

[58] Hajji S., Turki T., Boubakri A., Ben Amor M., Mzoughi N. (2017). Study of cadmium adsorption onto calcite using full factorial experiment design. Desalin. Water Treat..

[59] Preite M. (2019). Interpretación de Espectros Infrarrojos—Tabla de Espectroscopía Infrarroja QPG 3310—2do Semestre 2019.

[60] Ricci C., Miliani C., Brunetti B.G., Sgamellotti A. (2006). Non-invasive identification of surface materials on marble artifacts with fiber optic mid-FTIR reflectance spectroscopy. Talanta.

[61] Bishop J.L., Lane M.D., Dyar M.D., King S.J., Brown A.J., Swayze G.A. (2013). Spectral Properties of Ca-sulfates: Gypsum, Bassanite and Anhydrite. Am. Mineral..

[62] Launer P.J., Arkles B. (2013). Infrared Analysis of Organosilicon Compounds: Spectra-Structure Correlations.

